# Analyzing the contributions of transdisciplinary research to the global sustainability agenda in African cities

**DOI:** 10.1007/s11625-021-01042-6

**Published:** 2021-10-14

**Authors:** Sokhna Thiam, Fati Aziz, Sandra Boatemaa Kushitor, Akosua Baah Kwarteng Amaka-Otchere, Blessing Nonye Onyima, Oghenekaro Nelson Odume

**Affiliations:** 1 African Population and Health Research Center, West Africa Regional Office, Dakar, Senegal; 2 Institute for Health Research, Epidemiological Surveillance and Trainings (IRESSEF), Dakar, Senegal; 3 Department of Epidemiology and Public Health, Swiss Tropical and Public Health Institute, Basel, Switzerland; 4 Department of Ecology and Conservation Biology, Texas A&M University, College Station, TX 77843 USA; 5 Food Security Initiative and Centre for Complex Systems in Transitions, Stellenbosch University Stellenbosch, Stellenbosch, South Africa; 6 Department of Planning, Kwame Nkrumah University of Science and Technology, Private Mail Bag, Kumasi, Ghana; 7 Department of Sociology/Anthropology, Nnamdi Azikiwe University, PMB 5025, Awka, Nigeria; 8 Unilever Centre for Environmental Water Quality, Institute for Water Research, Rhodes University, P. O. Box 94, Makhanda, 6140 South Africa

**Keywords:** 2030 Agenda, Contribution of transdisciplinary approach, Sustainability, Synergies and trade-offs in SDG interactions

## Abstract

It is almost 6 years since the UN’s Sustainable Development Goals (SDGs) were adopted, and countries have less than 10 years to achieve the set targets. Unlike most of the world, sub-Saharan African countries have reported only minimal progress, one that the COVID-19 pandemic has unfortunately disrupted. Transdisciplinary research (TDR) has been conceptualized as important for achieving sustainability goals such as the SDGs. In this paper we (i) analyze the contributions of the five TDR projects toward the achievements of the SDGs at the city level in Africa, and (ii) explore the interactions between the assessed SDGs across the five projects. The projects’ contributions towards the achievements of the SDGs were examined in three thematic areas: (i) contexts, (ii) processes and (iii) products. The five projects were funded under the Leading Integrated Research for Agenda 2030 in Africa (LIRA) programme. The projects were being implemented in nine cities across five African countries Accra (Ghana), Kumasi (Ghana), Korhogo (Ivory Coast), Abuja Metro (Nigeria), Mbour (Senegal), Cape Town (South Africa), Nelson Mandela Bay Metro (South Africa), Grahamstown (South Africa) and Kampala (Uganda) and data were collected on each of the five projects in these cities. The contextual contributions include co-analysis and reflection on policy and institutional silos and social innovations amenable to contextual complexity. A shift in how actors perceived and conceptualized sustainability challenges and the role of the projects as transformative social agents constituted the two main process contributions. Tool development, virtual models and maps, and handbook are the product contributions by the projects. Our analysis of the SDG interactions indicated the need for cross-sectoral collaborations to ensures resource use efficiency, knowledge and experience sharing, and seamless flow of information and data to accelerate the SDG implementation.

## Introduction

Five years into the Sustainable Development Goals (SDGs) journey, the world is not on track to achieve the set goals (United Nations 2020b). With only minimal progress made, Sub-Saharan Africa countries are lagging in implementing these global goals (Sachs et al. 2017). The COVID-19 pandemic presents another threat to the SDGs, negatively impacting economies and societies worldwide (WHO 2020). The factors that inhibit achieving the global goals are multi-factorial, spanning structural, social, and economic domains (Jaiyesimi 2016). At the city level, governments are faced with multiple challenges such as rapid rise in urban population, employment, climate change, and environmental pollution that hinder the achievement of the SDGs (Patel et al. 2017). A concerted effort by all actors is, therefore, necessary to advance the achievement of SDGs. Stakeholder engagement has been emphasized when addressing the challenges limiting the SDG achievements (United Nations 2020a).

Transdisciplinary research (TDR) may accelerate the achievement of the SDGs because it is an inclusive research practice that draws on both academic and practice-based knowledge systems (Hansson and Polk 2018; Lawrence 2010). Recognizing the potential of TDR to contribute to sustainable urban development and the achievement of the SDGs in African cities, the International Science Council (ISC), through its Leading Integrated Research for Agenda 2030 in Africa (LIRA 2030) programme, has been providing support to twenty-eight (28) TDR over 5 years from 2017 to 2021.

TDR is understood as a knowledge co-production process with key stakeholders that generate formal, actionable knowledge on societal problems (Hadorn et al. 2006; Hadorn and Biber-klemm 2008; Lang et al. 2012). Working with different stakeholders helps researchers better understand local needs and interests, gain a holistic understanding of problems, and co-produce locally grounded knowledge and solutions (International Science Council 2020). Knowledge co-production makes SDG research issue-oriented rather than sector-focused and helps identify interactions and linkages across the different SDGs (International Science Council 2020).

TDR has been extensively theorized, particularly by scholars from the Global North (Hadorn et al. 2006; Hadorn and Biber-Klemm 2008; Hansson and Polk 2018). However, there is a growing body of knowledge on TDR from the Global South, including works by Thondhlana et al (2021), Breda and Swilling (2019), Culwick et al. (2019), and Patel et al. (2017). Theorization has aided in our conceptual understanding of TDR principles, project design, and processes. In literature, theoretical contribution has outpaced empirical studies in TDR (Lang et al. 2012). The implication is that much empirical research is needed to fully appreciate the contribution of TDR to addressing complex societal challenges. Such empirical studies would not only broaden our understanding of TDR but aid in its re-theorization. This paper is one of such studies that provide empirical evidence on the contributions of TDR towards the achievement of SDGs in nine African cities across six countries: Accra (Ghana), Kumasi (Ghana), Korhogo (Ivory Coast), Abuja Metro (Nigeria), Cape Town (South Africa), Mbour (Senegal), Nelson Mandela Bay Metro (South Africa), Grahamstown (South Africa) and Kampala (Uganda). The SDGs addressed are SDGs 2 (zero hunger), 3 (good health and well-being), 6 (clean water and sanitation), 7 (affordable and clean energy), 11 (sustainable cities and communities), and 13 (climate action).

The SDGs have been postulated as interlinked (United Nations 2015), implying that the pursuit of the achievement of one SDG ought to take into account its interaction and relationship with other SDGs. Interactions between SDGs can take diverse forms ranging from synergistic to trade-off interactions. Understanding the SDG interaction is very important for their achievements because it can lead to coherent and mutually reinforcing policies. It is, therefore, critical that in assessing the contributions of TDR projects towards the achievements of the SDGs, one takes into account the SDG interactions. Therefore the objectives of this paper are (i) to analyze the contributions of the five TDR projects toward the achievements of the SDGs at the city level in Africa, and, (ii) to explore the interactions between the assessed SDGs across the five projects. Three data collection templates were developed. The first template was on the research quality plus (RQ+) evaluative framework (McLean and Sen 2019; Ofir and Schwandt 2020). The second was on the contribution of TD research to the SDG achievements, and the third was on SDGs interactions*.* The projects’ contributions towards the achievements of the SDGs were examined in three thematic areas: (i) contexts, (ii) processes and (iii) products. We first used the RQ + evaluative framework to assess the projects’ research quality and impact before the contributions of the project to the SDGs were analyzed.

## Analytical approaches and methods

### Projects description and summary

The five TDR projects used in this study were among twenty-eight (28) collaborative research projects under the LIRA 2030 program. To support the grantees to contribute to solution-oriented, contextualized, and policy-relevant knowledge on the SDGs in African cities, the LIRA programme offers a series of capacity-building training workshops for all grantees throughout the 2-year project period in addition to the financial support it provides. We selected the five projects analyzed in this study because of the diversity and complementarity of the SDGs they were addressing and also because they were being implemented across multiple regions of Africa: west, east and south. Further, the projects were being implemented in cities of varying sizes, e.g. Cape Town (large city), Mbour (small city). We believe that the diversity of SDGs and the cities would allow a deeper analytical reflection on the contributions of the TDR projects towards the achievements of the SDGs. The projects started in May 2019 and will end at the end of September 2021. Details of each of the projects are presented in Table 1.

**Table 1:** Details of the five-LIRA projects

	Project 1 (2019–2021)	Project 2 (2019–2021)	Project 3 (2019–2021)	Project 4 (2019–2021)	Project 5 (2019–2021)
Project time frame and title	Climate change and diarrhoeal diseases in urban context: an integrated approach for sustainability in West African medium-sized cities (P1, climate change and diarrhoeal diseases)	Enhancing sustainability and resilience of Accra (Ghana) and Kampala (Uganda) through a water-energy-food Nexus approach (P2, ensureWEF)	Inclusive metabolism: using the co-produced theory of informal decentralized urban infrastructures to transform the delivery of urban food, water, and energy services in Ghana and South Africa (P3, informality and food systems)	Enhancing urban wetland and river ecosystem health (P4, urban river health)	Household energy use practices and potential interventions for sustainable consumption (P5, energy)
Objectives and short description	This project aims to understand the interlinkages between climate change and diarrhoeal diseases in urban communities to improve their management and to contribute to strengthening the resilience of health systems and the communities in the face of climate change. The approaches and methods used include community participation in the research process and implementation, climate and health data assessment, household surveys, key informal interviews	This project applies the Water-Energy-Food-Nexus approach to explore the status and governance of water, energy, and food resources for enhanced resilience of Accra and Kampala cities. The approaches and methods used include co-design and co-production workshops with key stakeholders in the WEF sectors, key informant interviews, household surveys, and inductive scenario development	This project aims to examine how informal infrastructure systems facilitate service provision using food systems as an entry point. More specifically, the project examines the movement of energy, water, and food, into, out of, and within Cape Town and Kumasi using mixed-method research. The methods adopted include participant observations, expert interviews, laboratory analysis, workshops, and photovoice	This project aims to recommend ways in which the health and functionality of urban rivers and wetlands can be enhanced to support sustainable urban development through the supply of valued and desired ecosystem services. The project proposes a systemic-relational (SR) ethically grounded approach within the complex social-ecological system framework as an analytical perspective for investigating the ecological, economic and social as well as management and institutional dimensions of urban rivers and wetland health. The approach departs from the traditional assessment as it recognizes that ecological and social-economic components together form an integrated and dynamic complex system of urban ecosystem health	The project aims at examining how informal infrastructure systems facilitate service provision using food systems as an entry point in Ghana and South Africa. The project considered the energy use practices households, factors influencing those practices, and potential interventions for promoting energy savings. The methodology includes workshops, questionnaire administration, institutional interviews, telephone discussions, and observation
Countries (cities) involved and host institutions	Senegal (Mbour), Côte d’Ivoire (Korhogo) Institut de Recherche en Santé, de Surveillance Épidemiologique et de Formation (*IRESSEF*)—Institute for Health Research, Epidemiological Surveillance and Training, Dakar, Senegal	Ghana (Accra), Uganda (Kampala) Council for Scientific and Industrial Research -Water Research Institute (CSIR-WRI), Accra, Ghana	Ghana (Kumasi), South Africa (Cape Town), Stellenbosch University, South Africa	South Africa (Nelson Mandela Bay Metro), Nigeria (Federal Capital Territory) Rhodes University, South Africa	South Africa (Makhanda-Grahamstown), Ghana (Kumasi) Rhodes University, South Africa
Key stakeholders involved	Mbour and Korhogo: National Government Ministries/departments and agencies, city/municipal and health authorities, academics, NGOs, local community leaders and community health workers	Accra and Kampala: National Government Ministries and agencies, city/municipal authorities, academic and research institutions, environmental consultancy firms, Not-for-profit (NFP) organizations, and residents of Accra and Kampala	Kumasi and Cape Town: city officials (metropolitan and municipal assemblies), food system actors, communities in Kumasi and Cape Town, and photographers	Nelson Mandela Bay and Federal Capital Authority: national government ministries/departments and agencies, city/municipal authorities, academics, NGOs, and local community members	Kumasi and Makhanda-Grahamstown: metropolital and municipal assemblies, National Government Agencies, public electricity utilities, academic and research institutions, local residents in the cities, and civil society organizations
SDG	SDGs 3 (good health and well-being), 6 (clean water and sanitation), 11 (sustainable cities and communities), and 13 (climate action)	SDGs 2 (zero hunger), 6 (clean water and sanitation), 7 (affordable and clean energy), and 11 (sustainable cities and communities)	SDGs 2 (zero hunger) and 11 (sustainable cities and communities)	SDGs 6 (clean water and sanitation) and 11 (sustainable cities and communities)	SDGs 7 (affordable and clean energy) and 11 (sustainable cities and communities)

### Data collection

The authors played active roles in each of the projects discussed, serving as principal or co-investigator or active team members in our respective projects. We collected data/reflected on the baseline situation of our projects using three templates/questionnaires we developed. The first template was on the RQ+ evaluative framework (McLean and Sen 2019; Ofir and Schwandt 2020), the second template was on the contributions of TDR projects toward the SDG achievements, and the third template was on the SDG interactions. For a common understanding, we interactively interrogated the collected data through online workshops. The data collection processes are described fully in the sections below.

#### *Data collection and RQ*+ *assessment*

The RQ+ assessment framework provides a systems-informed and transparent approach to defining and evaluating research quality and the positioning of the research for use and impact (McLean and Sen 2019; Ofir and Schwandt 2020). We assessed the quality of the projects using a data collection template/questionnaire (Appendix: Table 5) developed with insights from the RQ+ assessment framework. The template has two aspects: (i) contextual factors (key influencers of the research), and (ii) research quality dimensions (and sub-dimensions).

**Fig. 1:**
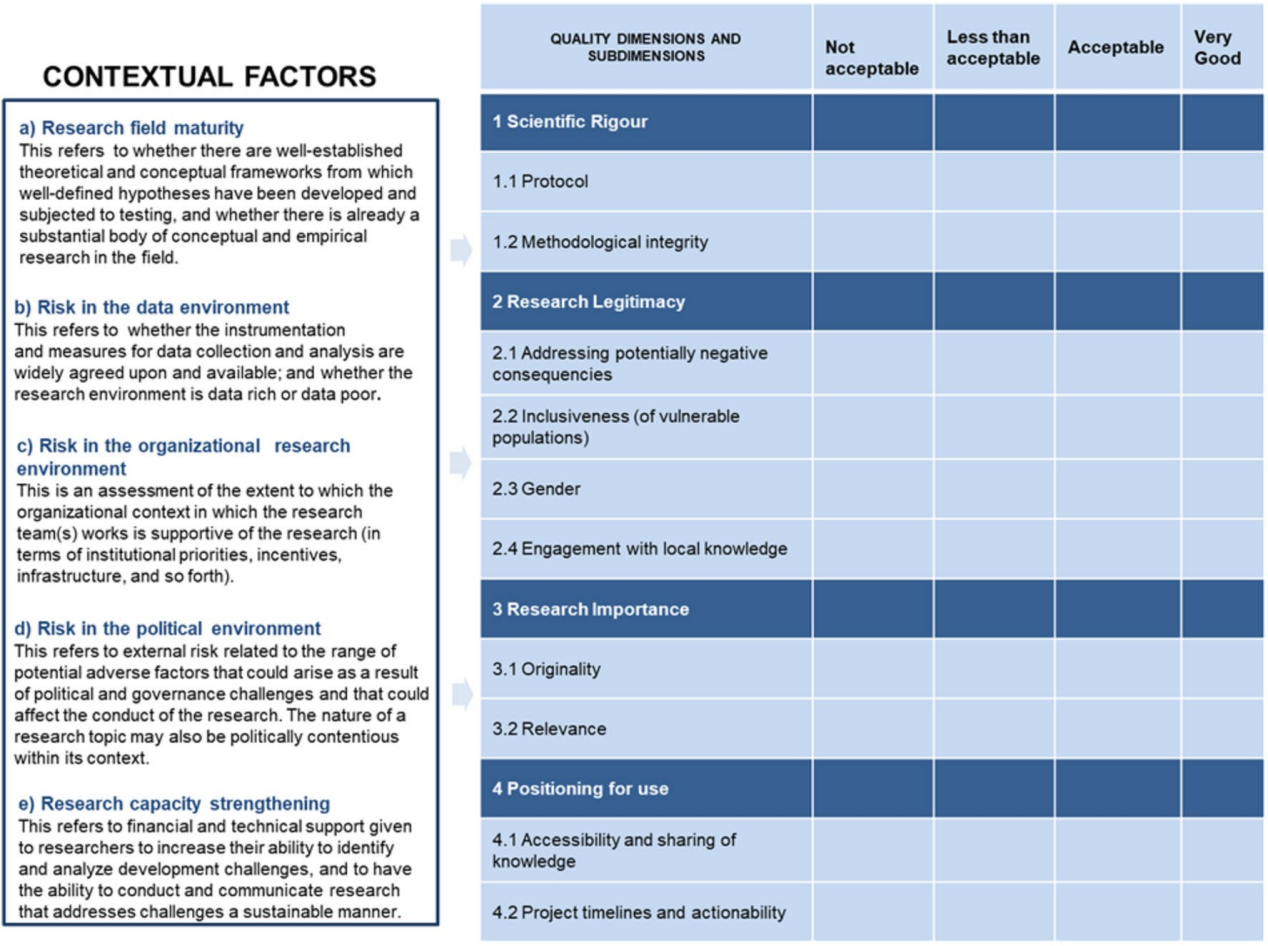
The contextual factors and research quality dimensions (and sub-dimensions) Adapted from Ofir and Schwandt 2020

The contextual factors are the issues within or outside the research environment that have the potential to affect the quality of research (either positively or negatively) and include: (a) maturity of the research field, (b) risk in the data environment, (c) risk in the organizational research environment, (d) risk in the political environment, and (e) research capacity strengthening. The research quality dimension (and sub-dimensions) of the RQ+ framework are: (a) scientific rigour, (b) research legitimacy, (c) research importance, and (d) positioning for use. Figure 1 shows the contextual factors and the research quality dimensions (and sub-dimensions) of the RQ+ assessment framework. A detailed description of the RQ+ assessment framework is presented in Ofir and Schwandt (2020).

After the data collection, we assessed the quality of the projects following two steps. In the first step, the key contextual factors: (a) maturity of the research field, (b) risk in the data environment, (c) risk in the organizational research environment, (d) risk in the political environment, and (e) research capacity strengthening, for each project were rated using a 4-point scoring system (as shown in Appendix: Table 6) based on the feedback received from the research team on the individual projects.

Regarding maturity of the research field, a score of 1 was awarded to a project if the field is considered matured. A score of 2 was awarded when the field was deemed to be established, 3 when it was an emerging one, and 4 when it was new. On risk in the data environment, a score of 1 was assigned to a project when the data environment posed no risk (i.e. there is an abundance of data in the project field). A score of 2 was awarded to a project when the data environment posed little risk to the project (i.e. data in the field of research is developed), 3 for data environment that posed a moderate risk to the project (i.e. fields with limited data), and 4 for projects operating in the field where the environment posed a high risk to the project (i.e. the field of research has weak data environments).

Regarding risk in the organizational research environment, projects with an empowering research environment scored 1; projects scored 2 when the environment was a supportive one, 3 when it was an unsupportive environment, and 4 when it was restrictive. On risk in the political environments, projects conducted in a stable political environment scored 1, a score of 2 was assigned to projects conducted in a moderately stable political environment, 3 for projects in an unstable environment, and 4 for projects in a volatile environment. On research capacity strengthening, projects where research capacity strengthening was of high focus scored 1, projects scored 2 when the focus was significant, 3 when the focus was limited, and 4 when the focus was low.

The second step involved evaluating the research quality of the projects (Ofir and Schwandt 2020). Given that the selected projects were at various stages of implementation with none completed yet, not all the research quality sub-dimensions of the RQ+ assessment were considered in the evaluation (see Table 5). For the dimensions scientific rigour, only sub-dimension protocol was evaluated. All the sub-dimensions of research legitimacy were assessed. These sub-dimensions are (i) addressing potentially negative consequences, (ii) inclusiveness of vulnerable populations, (iii) gender, and (iv) engagement with local knowledge.

Similarly, all the sub-dimension on research importance were also evaluated. These sub-dimensions are (i) originality and (ii) relevance. For the dimension on positioning for use, only sub-dimension knowledge accessibility and sharing were evaluated.

The rating of the projects’ dimensions/sub-dimensions (quality) was done with an 8-point scale (as shown in Table 6) based on the project aims and the extent to which the methods/activities stated therein were reflected on the ongoing projects. In addition to the response provided in the data collection sheet, additional information was obtained from the research proposals of the team and through several online discussions. Scoring was first done at the sub-dimension level and then aggregated to arrive at an overall score for each dimension. The scores for sub-dimensions of scientific rigour and positioning were recorded directly without any aggregation because they were the only sub-dimensions considered. A score of 1 to 2 indicated unacceptable level of achievement, scores of 3 and 4 indicated less than acceptable, scores of 5 and 6 indicated acceptable/good and scores of 7 and 8 revealed a very good level of achievement, as shown in Table 6.

#### Data collection and assessment of project contributions to the SDGs

We assessed the projects’ contributions in terms of (i) contexts, (ii) process, and (iii) products. For example, a contribution that alters how actors implement the SDGs or an even better understanding of the SDGs would qualify as a process contribution. Whereas when a project provides insights into the role of context on the achievement of the SDGs, such a contribution would be eligible as a shift in context. A product-related contribution was defined as a tangible outcome/product or modification of an existing product in light of SDG implementation. Contributions were conceptualized as theoretical and concrete. The template for collecting the project's data on contribution was developed to reflect the three identified aspects of research contributions towards achieving the SDGs. Each of the investigators (see Table 1) populated the template under the three contribution domains (themes) by responding to specific probing questions (Table 2). Each project investigator was also asked to reflect on the baseline situation before the project implementation and the enablers and constraints experienced during the project implementation.

**Table 2:** Data collection template on projects contributions to the SDGs supplied to the principal (co-) investigators for completion

Dimension	Baseline	Contribution	Constrain (inhibitors)/enablers
Context	Describe the baseline condition of the project context, focusing on (i) the actors (ii) social-ecological context (iii) cultural context (iv) economic and political context	What was the contribution to the context e.g., a shift in the understanding of the social-ecological context?	What were the specific factors that contributed to the success of your project and/or constrained the project from making meaningful contributions towards the SDGs?
Process	How were stakeholders in the project’s research area implementing the SDGs? E.g., a monthly collection of specific data related to the SDGs, quarterly inter-ministerial meetings How did the stakeholders conceptualize the SDGs and their main drivers in the project implementation environment?	What change was observed regarding ways in which stakeholders conceptualized and went about any aspects of the SDGs implementation due to project intervention? Note: change could be a shift in stakeholder, adapting data collection process due to project intervention, or any process-wise changes due to project intervention	Describe the factors that contributed to achieving these contributions or that constrain them
Product	Describe the baseline condition of the product Products are usually the tangible output in place before your research in relation to the specific research areas of interest. They can come in a variety of forms e.g., metrics for calculating energy consumption or monitoring the spread of disease, or a tool for monitoring water quality or scoring water quality change	Describe if your project has contributed to developing a new product or altering an existing product. Be specific in describing the contribution How does the product contribute to achieving the SDG?	Describe the factors that contribute to or constrain the product’s development or alteration?

The data provided on the baselines, contributions, enablers, and constraints were thematically analyzed following Braun and Clarke (2006). Project data were coded under major and sub-themes as deductive codes (Braun and Clarke 2006). A reflective and iterative process was followed to identify specific project contributions under the main domains/themes. When reflecting on project contributions within each domain, attention was paid to the sustainability, economic, social, and environmental dimensions.

#### Data collection for the SDGs interactions

The template for the data collection on the SDG interactions was based on the Nilsson framework for analyzing SDG interactions (Appendix: Table 7). The six (6) SDGs and twenty-two (22) targets and indicators addressed by the five projects are shown in Table 3. Using the templates, each of us supplied data on our project’s SDG targets, indicators, type of interaction and motivation for assumed interaction.

**Table 3:**
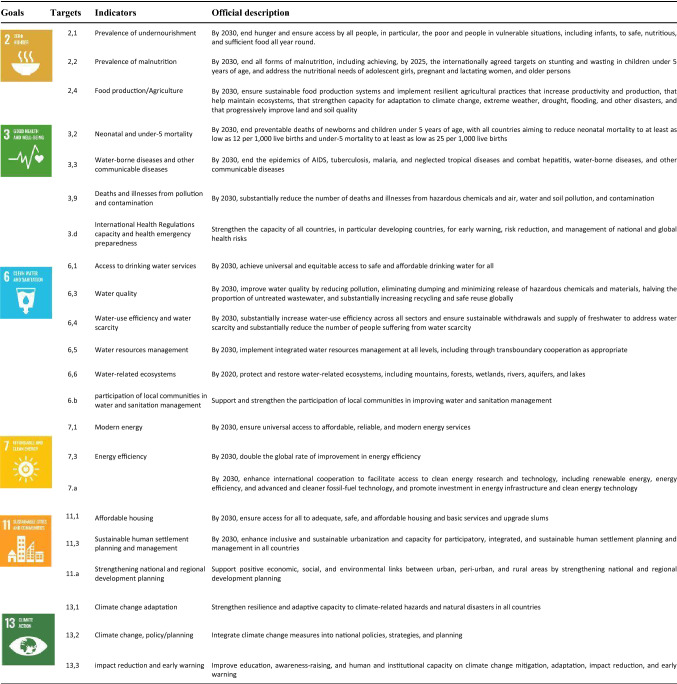
SDG targets and indicators addressed by the five LIRA projects in nine African cities

The seven-point scale framework developed by Nilsson et al. (2016, 2018) was used to assess the SDGs interactions because this framework was found to be particularly useful and robust. The framework not only differentiates between positive and negative interactions but also speaks to the strengths and directions of the interactions, thus making it robust to assess implications of SDGs inter-linkages at the city level beyond binary simplification of trade-offs and synergies (co-benefits). The seven-point scales are (i) indivisible (+ 3), (ii) reinforcing (+ 2), (iii) enabling (+ 1), (iv) consistent (0), (v) constraining (− 1), (vi) counteracting (− 2), and (vii) cancelling (− 3) (Nilsson et al. 2016, 2018). The first three interaction types are synergistic, whereas the remaining three interaction types are trade-offs. The signs depict the strength of the interaction on both types of interaction types, i.e. for the synergistic interaction, + 3 is the strongest, whereas − 3 is the strongest on trade-off interaction types. The interactions between the SDG targets addressed by the five projects were scored according to the seven-point system.

## Results

The results of the RQ+ assessment are first presented, followed by the contribution of the projects towards the achievement of the SDGs, including a presentation of the SDG interactions.

### *RQ* + *dimensions and key contextual influences on projects*

**Fig. 2:**
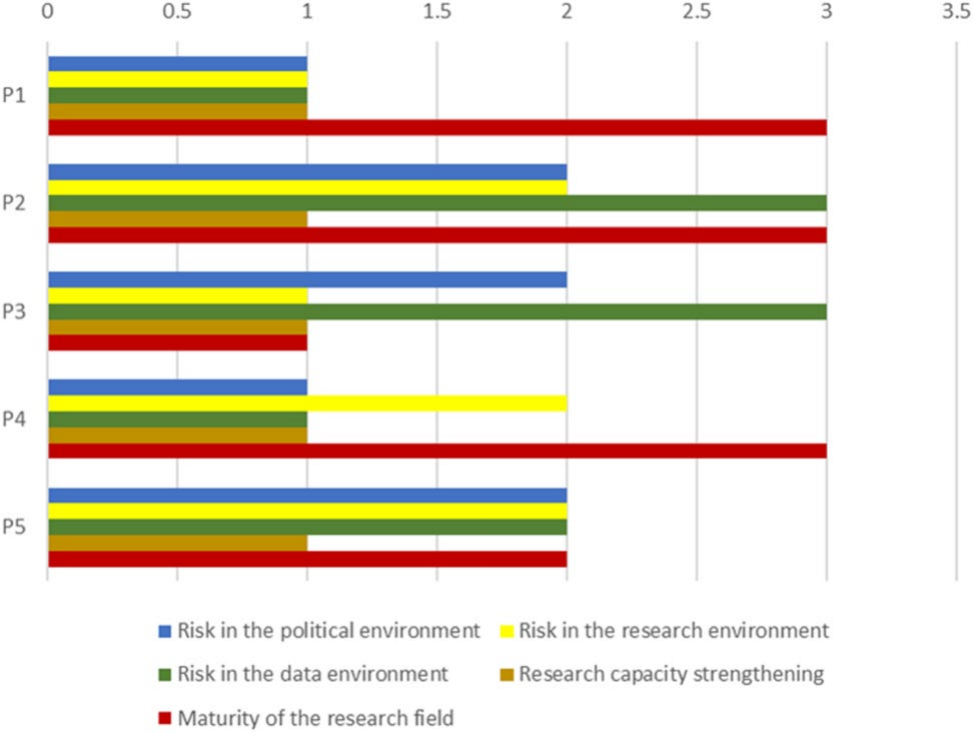
Results of the RQ+ key influencers

An examination of the key influences (contextual factors) on the five projects indicated that most of the projects (3 out of 5) are in emerging fields and have a strong focus on research capacity strengthening and with the majority (3 out of 5) of them being conducted in supportive organizations, and institutions (Fig. 2).

Based on the dimensions (and sub-dimensions), the research quality analysis of the projects showed that the five projects were of acceptable/good to very good quality (Table 4). All the projects achieved very good scores in the dimension positioning for use and research importance. For the dimensions of scientific rigour and research legitimacy, the projects fell in the acceptable/good category.

**Table 4:** Scores of RQ+ dimensions and sub-dimensions across the LIRA 2030 projects

RQ+ dimensions	P1	P2	P3	P4	P5
1. Scientific rigour	**6**	**6**	**6**	**6**	**6**
1.1 Protocol	6	6	6	6	6
2. Research legitimacy	**6.3**	**6.0**	**5.8**	**5.8**	**5.8**
2.1 Addressing potentially negative consequences	5	5	6	5	5
2.2 Inclusiveness	7	6	6	7	7
2.3 Gender responsiveness	6	6	4	4	4
2.4 Engagement with local knowledge	7	7	7	7	7
3. Research importance	**7**	**7**	**7**	**7**	**7**
3.1 Originality	6	6	7	6	6
3.2 Relevance	8	7	7	8	8
4. Positioning for use	7	7	7	7	7
4.1 Knowledge accessibility and sharing	7	7	7	7	7

The RQ+ assessment indicated that two of the projects, P2 (Ensure WEF, Ghana, and Uganda) and P3 (informality and food systems, Ghana and South Africa), showed a moderate risk in the data environment. Owing to the multidisciplinary nature of P2 (Ensure WEF, Ghana, and Uganda), large and diverse datasets from multiple areas (water, energy, food) were needed for the research analysis. Given that relatively few studies had been conducted in the region on WEF nexus, the research team had to engage with the diverse stakeholders to acquire datasets. The lack of adequate quantities or types of data for WEF nexus research has been noted as a significant limitation by many (Hurford and Harou 2014; Semertzidis 2015; Wolfe et al. 2016). For this reason, the WEF project scored moderately on the risk the data environment posed to the project. A systematic review of existing WEF nexus methods revealed that discussions on specific methods to evaluate the WEF nexus are emerging and rapidly growing (Albrecht et al. 2018).

In the case of P3 (informality and food systems, Ghana and South Africa), the moderate risk associated with the data environment is attributed to the complexity of African informality and food systems. For example, researchers working in this area must consider all the activities involved in the food chain and the role of informality in accelerating nutrition security. Although various methods exist for analyzing different aspects of the food system, systems modelling techniques have been adopted in the informal food sector (Fuseini et al. 2018). Such system modelling techniques are few and currently being developed (Global Panel 2017), supporting the RQ+ assessment, which placed this project as being implemented in a moderate-risk data environment.

The assessment of P5 (Energy, Ghana and South Africa) showed that data in the research field is developed. The methodology and instruments for collecting both quantitative and qualitative data for the research are available and widely agreed upon. The instruments designed consisted of questionnaire interviews for household surveys, quantitative data collection from institutions, key informant interviews and group discussions. However, the research team indicated that although the environment is data-rich, access to institutional data is besetted with barriers.

Projects P1 (Climate change and diarrhoeal diseases, Senegal and Ivory Coast) and P4 (Urban River Health, South Africa and Nigeria) showed a flourishing data environment. In P1 (Climate change and diarrhoeal diseases, Senegal and Ivory Coast), a District Health Information System (DHIS2), a platform for collecting and storing health data, exists in the two countries. The countries’ national Meteorological and Weather Services have existing climate data to which the research team were given access. As diarrhoea is a disease with several drivers, there are methods for analyzing different aspects of the diseases, and method to predict and estimate diarrhoea risk under climate change is currently developed (WHO 2014; Kolstad and Johansson 2011). In P4 (Urban River Health, South Africa and Nigeria), the instrumentation and methodology for the data collection methods adopted in the research (e.g. physicochemical and microbial measurement of the rivers) are widely agreed upon and available. An abundance of data sources also exists; therefore, the research team had no difficulties assessing the data.

Given the rigorous project selection procedure adopted by LIRA, and the additional trainings on proposal development provided to the researchers, it is not surprising that the ratings for the different dimensions across the five projects ranged from acceptable/good to very good. Once the call for project proposals were closed, the LIRA scientific committee pre-selected a number of projects and invited the project PIs to a 5-day training designed to ensure that the researchers receive the necessary skills and capacity for inter-and-trans-disciplinary research during the 2-year project cycle. The period was also used for developing and fine-tuning the project proposals for the second round of scientific review and selection.

The projects are by their nature transdisciplinary and were co-designed and co-implemented with policy and societal actors. The deep involvement of the societal and policy actors suggest a high probability of the stakeholders’ uptake of the research products once completed. A deep stakeholder engagement enhances research uptake in policy and practice (Phillipson et al. 2012).

### Projects contribution to the SDGs

#### Context contributions

The analysis of the different projects suggests that the policy, social, economic, ecological, and institutional contexts were the main defining factors shaping the implementation of the SDGs at the city level. The contextual contributions of the analyzed projects can be summarised as (i) co-analysis and reflection on policy and institutional silos, and (ii) social innovations amenable to contextual complexity.

*Co-analysis and reflection on policy and institutional silos *In nearly all the projects’ implementation environments, key policy and institutional actors were found to be pursuing their agenda in silos, with little or no cross-sectoral collaborations. For example, in P4 (Urban river health, South Africa and Nigeria), key government ministries (e.g., Ministries of Environment and Water Resources in Nigeria), departments (e.g., Department of Environmental Impact Assessments in Nigeria), and agencies (e.g., Abuja Environmental Protection Board, Nigeria; and the National Environmental Standards Regulatory and Enforcement Agency in Nigeria), which are responsible for environmental and water resources management and governance were found to be pursuing their agenda in silos. For P2 (Ensure WEF, Ghana, and Uganda), key policy and institutional actors in the water, energy, and food sectors were also found to be working in silos, despite empirical evidence supporting the interlinkage of the WEF nexus. For P1 (Climate change and diarrhoeal diseases, Senegal and Ivory Coast), key government ministries (e.g. Ministry of Health, Water and Sanitation, and Environment) and agencies (e.g. National Agency for Civil Aviation and Meteorology of Senegal) do not regularly share data among themselves, suggesting silos. We found that the Ministry of Health does not readily have access to water and climate-related data to determine the connection between diarrhoea cases and water quality and climate change. To address the inherent silos observed at the beginning of the projects, co-design processes and knowledge co-production workshops facilitated co-analysis and reflection on the importance of cross-sectoral collaboration for the SDGs. The co-design and knowledge co-production processes contributed to shifting actors’ insights and understanding of the complexity of the SDGs interlinkages and the importance of integrated, systemic policy frameworks for the SDGs. The co-design and knowledge co-production processes also created momentum on the imperative for establishing concrete mechanisms/actions for breaking policy and institutional silos. For example, in P2 (Ensure WEF, Ghana and Uganda), actors from the Ghana Ministry of Energy, Ministry of Food and Agriculture, Ministry of Water Resources, Works, and Housing participated in project co-design and knowledge co-production workshops, providing opportunities for actors to transcend institutional and sectoral silos. Similar workshops in P5 (Energy, Ghana, and South Africa) involved actors from the Energy Commission, the Electricity Company of Ghana, the Civil Society, and the Metropolitan, Municipal, and District Assemblies. Also, in P1 (Climate change and diarrhoeal, Senegal and Ivory Coast), the knowledge co-production workshops involved actors from the Ministry of Health, Water and Sanitation, and Environment. The project shed light on the criticality of multi-sectoral collaboration and data sharing through the workshops to break institutional silos. Given the limited timeframe of the project implementation, it is too early to assess the impact of these contributions to the achievements of the SDGs in the respective cities where the projects are being implemented. Nevertheless, the findings have implications for transcending policy and institutional silos, which have been implicated as key impediments to the achievements of the SDGs. This is particularly true as the importance of the interlinkages of the SDGs, and the imperative for cross-sectoral collaborations are taking the central stage on SDGs discussions (Nilsson et al. 2018).

**Fig. 3:**
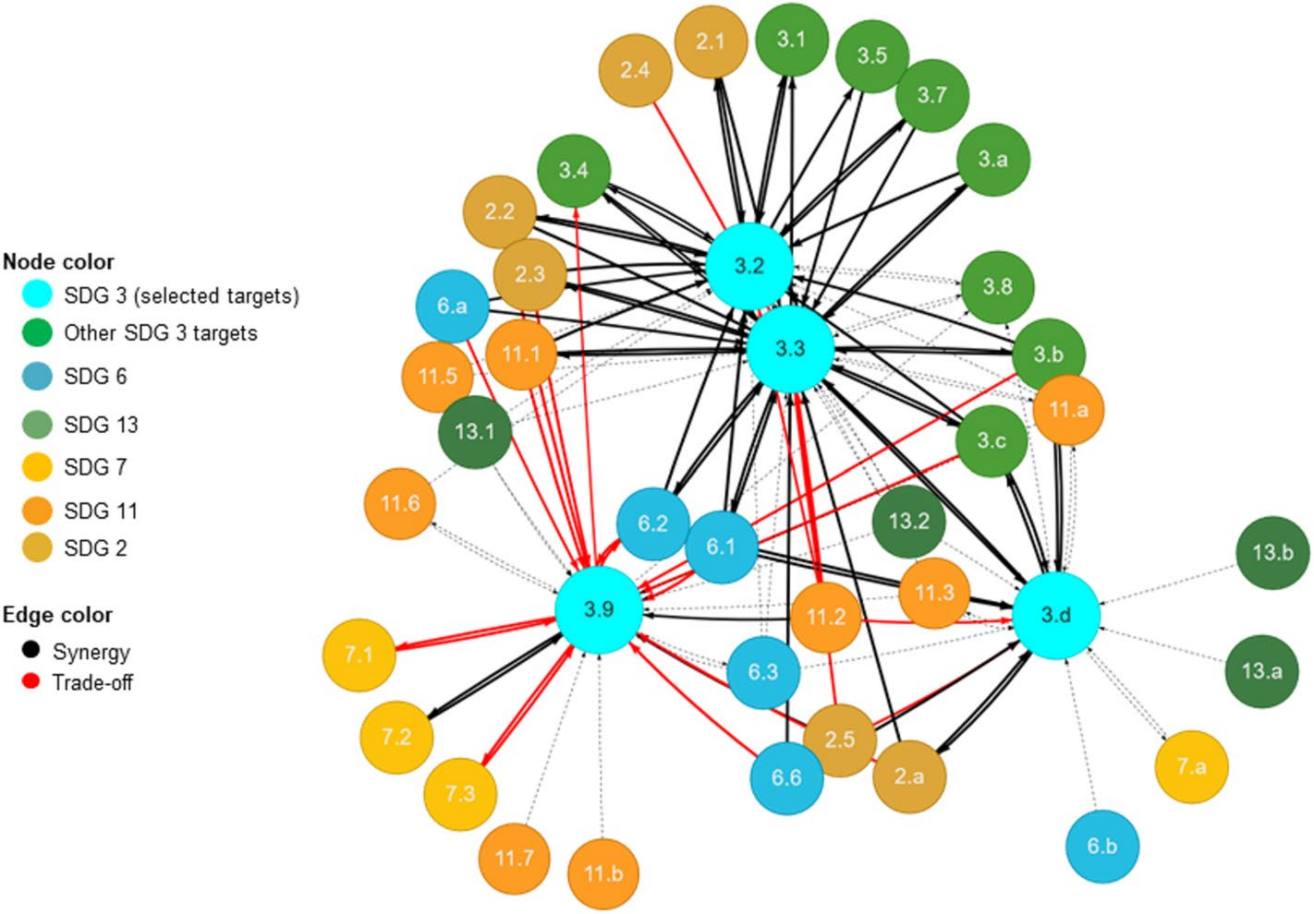
Results of network analysis: links between selected SDG 3 target indicators and other SDG target indicators addressed by the five ongoing LIRA projects

**Fig. 4:**
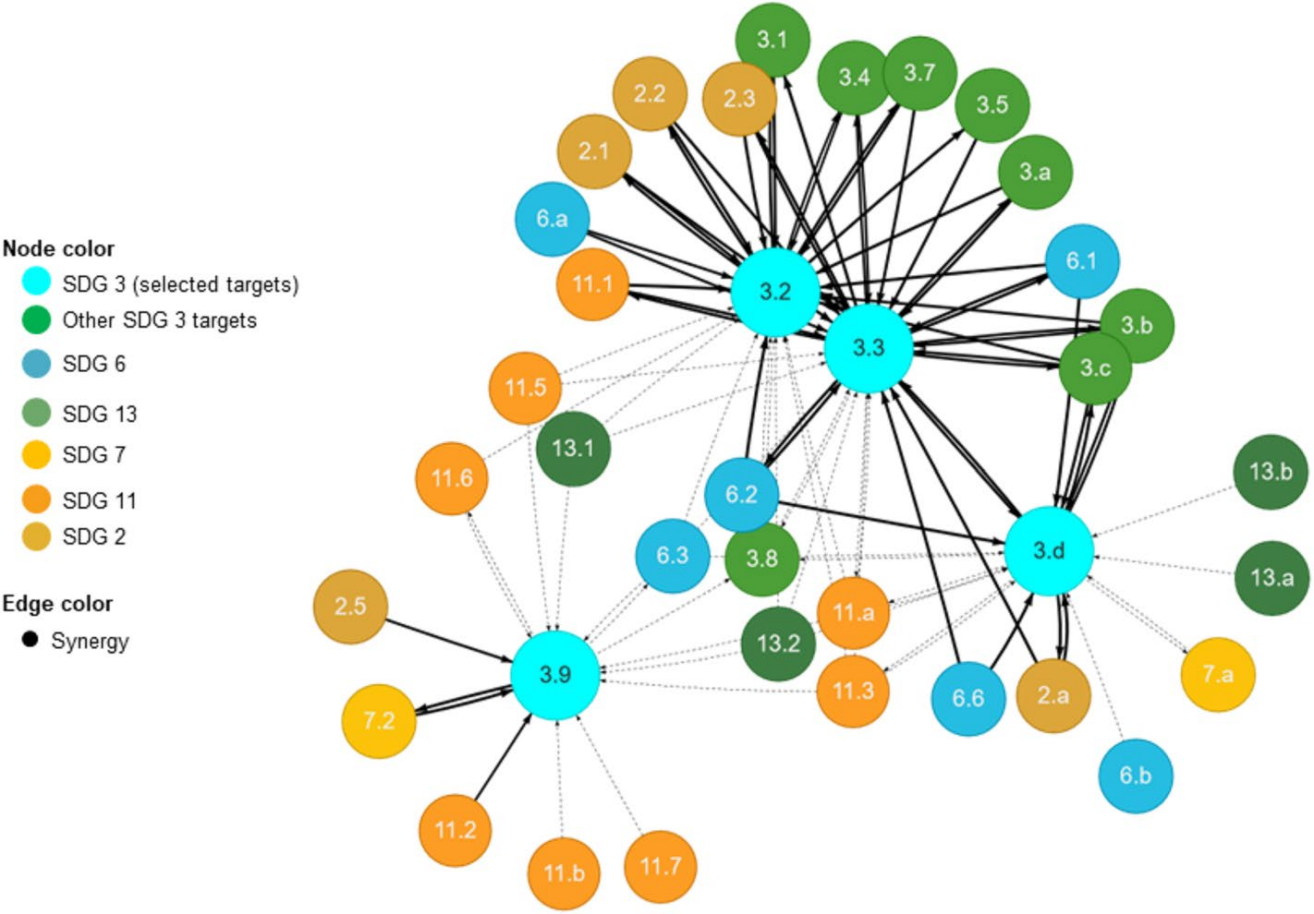
Results of a sub-network analysis of positive interactions

**Fig. 5:**
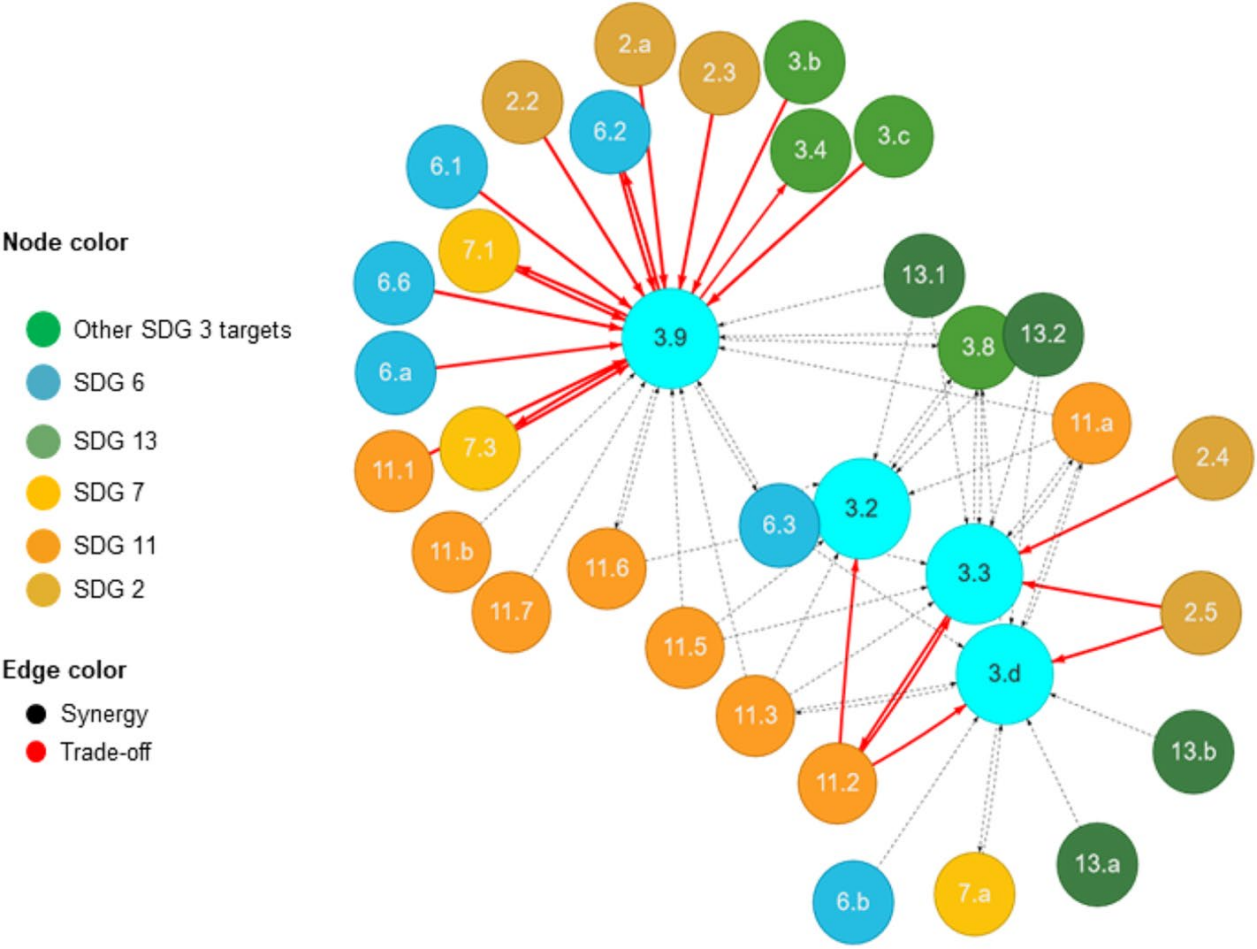
Results of a sub-network analysis of negative interactions

For example, the complexity of interactions between targets 3.2, 3.3 and 3.9 of SDG 3 and selected targets of the other SDGs provided in Fig. 3 illustrates the relevance of the SDGs interlinkages. The interactions were both positive (Fig. 4) and negative (Fig. 5). Concerning the positive interactions (Fig. 4) targets 3.3 (*water-borne diseases and other infectious diseases*), 3.2 (*neonatal and under-five mortality*), 3.9 (*deaths and illnesses from hazardous chemicals and air, water, and soils pollution and contamination*), and 3.d (*health risk management*) showed strong positive interactions with targets 2.1 (*hunger*), 2.2 (*malnutrition*), 2.3 (*agriculture productivity*), 6.1 (*access to drinking water*), 6.2 (*sanitation and hygiene*), 11.1 (*access to urban housing and essential services*) and 7.2 (*renewable energy*). The strong positive interactions between these SDG targets suggest the relevance of integrated, cross-sectoral policies that can lead to concurrent achievements of the SDG targets. For example, the strong positive interaction between SDG target 3.3, 2.4 and 6.1, 6.2 can be explained by the fact that (i) realizing nutrition security together with safe, equitable water and sanitation services can directly reduce neonatal and child deaths. Our analysis is supported by a household study conducted in the city of Mbour by Thiam et al. (2017), which found that only 59% of the surveyed households were connected to the water network system, with significant disparities between neighbourhoods. The study further indicated that 72% of the studied population empty their wastewater on the street, increasing the risk of drinking water contamination and thus diarrhoea risk among children (Thiam et al. 2017).

Regarding the negative interactions (Fig. 5), targets 3.9 (*reduce deaths and illnesses from hazardous chemicals and air, water, and soils pollution and contamination*) and 3.3 (*end water-borne diseases and other communicable diseases*) showed strong negative interactions with targets 2.2 (*malnutrition*), 2.3 (*agriculture productivity*), 6.1 (*access to drinking water*), 6.2 (*sanitation and hygiene*), 7.1 (*universal access to affordable, reliable and modern energy services*), 7.3 (*double rate of improvement in energy efficiency*) and 11.2 (*access to safe, affordable, accessible and sustainable transport systems for all*) (Fig. 5). The strong negative interactions between these SDG targets indicate the danger of single-mindedly pursuing a particular SDG without considering how other SDGs may affect or how it affects other SDGs. For example, the negative interaction between SDG target 3.9 and 2.2, 2.3 can explain that increasing nutritional security via conventional agriculture can improve soil and water pollution, constraining the reduction of deaths and illnesses caused by hazardous chemicals. Such chemicals can however adversely affect human health, particularly of newborns and children. Moreover, improving water quality reduces water-borne diseases and by extension, under-five child deaths resulting from diarrhoeal diseases. This could lead to unanticipated growth in population such that without the commensurate infrastructural provision, available health and water facilities might be constrained. This takes the city water management institutions back to where they started from—a rebound effect. Overall, the SDG analysis suggests the imperative for systemic, integrative and holistic policies that are sufficiently cross-sectoral, and intentionally avoiding the silo approach across scale and governance processes.

*Social innovations* Social innovation addresses social needs by creating new ideas, strategies, concepts, initiatives, and processes, impacting resource allocation, authority, and power (Westley et al. 2006). Sources of social innovations are diverse, including research projects, community groups, NGOs, governments, businesses, and researchers. According to Biggs et al. (2010) bricolage, contagion are the two dynamic processes involved in social innovation. Bricolage creates something novel by combining existing and new ideas, whereas the diffusion or dissemination of innovation is referred to as Contagion (Biggs et al. 2010). Incremental and radical innovations are the two categories of social innovations (Biggs et al. 2010). Incremental innovations are gradual, steady, and predictable, whereas radical innovation is more prone to failures and unexpected eventualities (Biggs et al. 2010). Key social needs in the P3 (Informality and food systems, Ghana and South Africa) implementation environment were (i) the imperative to assure nutrition security for school children (at the Ubunye Educare Centre) through the provision of quality food, (ii) provide nutrition education, and (iii) food gardens. Responding to the identified social needs, P3 (Informality and food systems, Ghana, and South Africa) mobilized the community agency to construct a prototype upcycled vertical garden, planting high-quality vegetables such as spinach, spring onion, Asian lettuce, mint, and thyme. Through the embedded social and institutional networks, the project aims to achieve contagion of this bricolage innovation across scales within the Western Cape Province of South Africa and Kumasi in Ghana. The food system innovation in P3 is regarded as radical as being implemented in school settings for the first time.

#### Process contributions

Processes play vital roles in the achievement of the SDGs. Often, processes lead to concrete outcomes over time. The analysis of the projects suggested two significant process-related contributions. These are (i) transformative social learning and (ii) transformative social agency and institutional entrepreneurship.

*Transformative social learning* As all the projects were intentionally designed as TDR projects, they actively brought together diverse actors to stimulate reflexive and critical engagement with the projects’ SDGs. We found that the process of spontaneous and critical engagement led to transformative learning about inherent tensions, complexities, and risks embedded in the SDGs. For example, P5 (Energy, Ghana, and South Africa) created an enabling environment that brought together researchers, individual energy consumers, government regulatory and supply agencies (i.e. Energy Commission of Ghana and the Electricity Company of Ghana). The project’s conducive multi-actor environment provided the impetus for reflexive, boundary-crossing learning and capability development to respond to the energy crisis, which the project sought to address. The project achieved a conducive multi-actor environment through co-identification of the research problem, co-design and co-implementation of interventions, underpinned by co-learning, adaptation, and constant reflections among the actors. In all projects, we found that expanded epistemological horizon through actor boundary-crossing, synergy, and hybridity contributed to a shift in how actors perceived and conceptualized the SDGs each project sought to address.

Our findings seem to implicate transformative social learning, which has been theorized as critical for transitioning the sustainability challenges that confront humanity (Macintyre et al. 2018). Transformative social learning has been argued to include contextually sensitive reflexive learning processes around uncertainties, risks, tensions, discontinuities across scale, and contexts (O'Donoghue et al. 2007). Lotz-Sisitka and colleagues identified reflexive social learning and capability theory as emerging transformative learning research and praxis within the field of sustainability science (2015). As our analysis indicated, reflexive social learning involves boundary-crossing, engaging diverse perspectives and insights to create the notion of hybridity and synergy that bring about transformative learning through dialogic, multi-loop engagement, and interactions.

*Transformative social agency and institutional entrepreneurship* The analysis of the projects suggests that the embedding of transdisciplinary principles such as project problem co-identification, project co-design, co-implementation, and co-learning accelerated the mobilizing of transformative social agents and institutional entrepreneurs across the multiple contexts in which the projects were being implemented. For example, through such change agent and institutional entrepreneurs in P4 (urban river health, South Africa and Nigeria), actors across multiple government agencies, e.g. the Federal Ministry of Environment, Nigeria, the Nigeria Hydrological Services Agency, the Abuja Environmental Protection Board, Nigeria, National Environmental Standards Regulatory and Enforcement Agency (NESREA), Nigeria, Department of Water and Sanitation, South Africa, the Nelson Mandela Metropolitan Municipality, South Africa, etc., were mobilized for the protection of the urban rivers and wetlands health. Similarly, social agents identified as community champions in P5 (Energy, Ghana, and South Africa) were instrumental in fostering logic for experimentation in energy-saving practices and behaviour at the household level.

The findings of this study provided empirical evidence in support of the role of transformative social agents in perturbing systems for change (Wesley et al. 2013). Transformative agents and institutional entrepreneurs were able to mobilize resources, use their networks, skills, and knowledge to challenge system-wide, organizational and institutional norms, culture, and beliefs to catalyze solutions and changes to complex sustainability challenges.

#### Product contributions

The analysis of the projects revealed three product contributions towards the realization of the SDGs: (i) tool development, (ii) virtual models and maps, and (iii) handbook. Concerning tool development, P4 (Urban river health, South Africa and Nigeria) developed a macroinvertebrate-based index to monitor urban river health for Nigeria streams. Macroinvertebrates are known as excellent indicators of river health and are widely used globally for the bioassessment of rivers and streams (Fei et al. 2016). The development of the index can contribute to the protection of river resources (SDG 6) by applying the index in water quality licencing, assessment of biodiversity, and habitat integrity (Edegbene et al. 2020).

The second product—virtual models including maps and photos—was identified in the creation of mental models of the SDGs, their complexities, and interlinkages, which facilitates systemic thinking necessary for the SDG implementation. For example, P3 (Informality and food systems, Ghana and South Africa) created a virtual photo exhibition of resource flows in cities across Africa. The exhibition allowed city residents to tell their stories about water, food, transport, energy, and waste.

For the third product, a handbook and logbook were produced by P5 (energy, Ghana, and South Africa) using a participatory appraisal approach with written and graphical illustrations developed in English and Twi (the local language of the stakeholders) languages. Participants can refer to the handbook and logbook for co-designed energy savings and conservation techniques, which contribute directly towards the achievement of SDG 7.

## Conclusion and recommendations

In this paper, we analyzed the contributions of five TDR projects towards achieving the SDGs in African cities. We analyzed TDR projects’ contributions to the SDGs in terms of (i) contexts, (ii) processes and (iii) products. Contextual contributions include co-analysis and reflection on policy and institutional silos and social innovations amenable to contextual complexity. A shift in how actors perceived and conceptualized sustainability challenges and the role of the projects as transformative social agents constituted the two main process contributions. Our analysis of the projects revealed that (i) tool development, (ii) virtual models and maps as well as (iii) handbook are the significant product contributions by the projects towards the realization of the SDGs at the city level. Our analysis of the SDG interactions indicated the need for cross-sectoral collaborations. Such cross-sectoral collaboration can contribute to the SDG achievements in several ways. First, it ensures resource efficiency as multiple sectors share resources without duplication of efforts. Second, it provides knowledge and experience sharing on the successes and failures of implementing the SDGs. Third, it ensures seamless flow of information and data needed for the SDGs’ implementation.

## Appendix

See Tables

**Table 5:** Data collection template for assessing the projects’ contextual factors and quality dimensions (and sub-dimensions)

(i) Contextual factors (key influencers of the research)	
(a) Maturity of the research field	Describe the maturity of your research field using the following questions as a guide: Does your research field have well-established theoretical and conceptual frameworks from which well-defined hypotheses have been developed and subjected to testing? Does the field have a substantial body of conceptual and empirical research? How active is the research field? Are there many researchers in the field?
(b) Research capacity strengthening	What form(s) of support are you receiving from the project sponsors in order to increase your ability to conduct, manage and communicate your research over time and in a sustainable manner? Note: the support may be financial or technical
(c) Risk in the data environment	Are the instrumentation and measures for data collection and analysis in your research area widely agreed upon and available? What about the data for the research itself? Is it available and assessable? Please indicate if the research environment data rich or otherwise?
(d) Risk in the research environment	Is your organization (institution) supportive of the research? Please elaborate on the forms/types of support being provided Note: “supportive” refers to institutional priorities, incentives, infrastructure, and trainings to support the research
(e) Risk in the political environment	Are there any potential adverse factors (such as electoral uncertainty, policy instability, political destabilization, a violent conflict, or a humanitarian crisis) that could arise because of political and governance challenges and that could affect the conduct of your research? Is the nature of your research topic politically contentious within its context?

(ii) Research quality dimensions (and sub-dimensions)		
Scientific rigour	1.1 Protocol	Please describe the steps you took to ensure methodological rigor. Consider issues such as validity, reliability and transferability or generalizability, and integration (in mixed methods design)
Research legitimacy	2.1 Addressing potentially negative consequences	What are the steps you took to address the risk of potentially negative consequences of your project? Please describe this in relation to the potential risks in the research processes and/or outcomes for affected or targeted populations Has the research been approved by an institutional or alternative research ethics board? Please provide evidence
	2.2 Inclusiveness of vulnerable populations	How did your project consider vulnerable populations? Were you inclusive in selecting research participants or potential beneficiaries?—not excluding anyone on the basis of culture, language, religion, race, economic status, disability, sexual orientation, ethnicity, linguistic proficiency or age? If not, kindly provide a reason for the exclusion Did you ensure that the interests of vulnerable, marginalized communities or populations are a priority? If not, kindly provide justification for the contrary
	2.3 Gender	How is your project designed to incorporate gender (e.g. in terms of data collection and analysis, engagement with stakeholder)? Is the project design sensitive to the needs and special situations or people of different genders?
	2.4 Engagement with local knowledge	Does your project engage communities, populations or stakeholders in an appropriate and credible manner, including indigenous and minority ethnic or social groups, and building their capacities where appropriate? Does it respect traditional knowledge, wisdom and practices, as well as local contexts, researchers and contributors to the research? Does it ensure appropriate benefits for stakeholders from their participation in the research process (e.g. access to research findings in appropriate formats and through appropriate processes)? How does your project address the identified needs and/or priorities, given the scale of the research?
Research importance	3.1 Originality	With reference to the current state of knowledge in your field, please describe (if any) the new insights and knowledge for theory and practice your project is contributing to
	3.2 Relevance	What informed the selection of your research topic? Please indicate whether the research objectives and research questions targeted at: Solving a problem that is a proven priority for key development stakeholders, and/or Aligning with key development policies, strategies and priorities, and/or Focusing on emerging problems that are likely to demand solutions in the near future
Positioning for use	4.1 Knowledge accessibility and sharing	Please describe your stakeholder engagement strategy. What is the extent to which to which your research findings, processes and products (a) are targeted to and engage user groups, (b) reflect an understanding of the contexts of potential users, and (c) match the ways potential user groups access and engage ideas and information (e.g., workshops, policy briefs for policymakers)

5,

**Table 6:** The research quality sub-dimensions used in the study, with their rubrics Apdated from Ofir and Schwandt 2020

Contextual factors			
Maturity in the research field			
(1) Mature field	(2) Established field	(3) Emerging field	(4) New field
Well-established and recognized theoretical and conceptual frameworks	-Theoretical and conceptual frameworks in development but generally recognized.	Research capacity strengthening was considered to a minimum in project strategy, but there were few activities dedicated to it	- Very limited theoretical or conceptual frameworks are being debated or rapidly changing and largely unrecognized
-A substantial body of conceptual and empirical research	-A body of conceptual and empirical research that reflects significant growth.	Research capacity strengthening was considered to a minimum in project strategy, but there were few activities dedicated to it	- Scarce empirical or theoretical body of research
-Discernible knowledge sharing outlets (journals, conferences, curriculum)	-Discernible knowledge sharing outlets (journals, conferences, curriculum)	Research capacity strengthening was considered to a minimum in project strategy, but there were few activities dedicated to it	- Few dedicated journals or academic programs
-A vibrant community of experienced researchers	- An ample community of active researchers who easily associate with the field, and are connected to each other	Research capacity strengthening was considered to a minimum in project strategy, but there were few activities dedicated to it	- Few active researchers are seeking to be recognized and connected

Research capacity strengthening			
(1) Strong focus	(2) Significant focus	(3) Limited focus	(4) Low focus
Research capacity strengthening was an explicit objective and counted as one of the priorities of the project. There were capacity-building activities throughout the project	Project design included research capacity strengthening explicitly (but not as a priority), and there were some activities related to it.	Research capacity strengthening was considered to a minimum in project strategy, but there were few activities dedicated to it.	Research capacity strengthening was not an objective, and no discernible

Risk in the data environment			
(1) Flourishing	(2) Developed	(3) Limited	(4) Weak
- Instrumentation and measures for data collection and analysis are widely agreed upon and available	- The necessary instrumentation and measures for data collection and analysis are generally available	- There are few instruments and measures for data collection and analysis available	- Instrumentation and measures for data collection and analysis are generally unavailable
- Body of data is well developed, stable and with significant open data resources	- Body of data has reasonable availability and is generally credible	- Limited quantities of data, and/or some credibility gaps	- Data scarcity and with lack of credibility
- Abundance of national and international data sources	- Diversity of international data sources, but few at the national level.	- Few international and national data sources	- Data sources are scarce.

Risk in the research environment			
(1) Empowering	(2) Supportive	(3) Unsupportive	(4) Restrictive
Research environment (organizational priorities, infrastructure, norms, incentives, etc. related to research) is fully established and enabling for researchers.	Research environment is well developed and generally supports researchers with their needs.	Research is not an organizational priority, yet the organization tends to comply with acquired commitments or external requests.	Research environment is weak or largely under-developed, not supportive of researchers or possibly even works against them.

Risk in the political environment			
(1) Stable	(2) Moderately stable	(3) Unstable	(4) Volatile
Stable political environment with solid governance practices, lack of significant social conflicts, and no personal risks to researchers	Generally stable political environment, with established governance practices, unusual major social conflicts, and no personal risks to researchers	Political environment that features some levels of instability and recurrent change, some major social	Very unstable or unpredictable political environment with weak governance practices, social conflict, and/or potentially significant risks to researchers

Research quality dimensions and sub-dimensions			
Dimension 1: Scientific Rigour			
Sub dimension 1.1: Protocol			
Unacceptable	Less than acceptable	Acceptable/Good	Very Good
1 2	3 4	5 6	7 8
#NAME?	- Research design was articulated but left some gaps.	- Research design was clearly articulated and transparent.	- Research design was clearly articulated, and the research protocol was open, and accessible where appropriate.
	- Adherence to methodological standards for the field was not fully established.	- Adherence to methodological standards for the field was established and largely achieved.	- Adherence to methodological standards was consistently demonstrated, and innovations were considered and introduced were appropriate.
	- Literature/document review was partially insufficient	- Literature/document review was appropriate and shows how the project contributes new/valuable knowledge (relevant, up-to-date, structured, etc)	- Literature/document review was appropriate and comprehensive, presenting the state of knowledge on the research topic and the importance of this particular contribution.

Dimension 2: Research Legitimacy				
Sub dimension 2.1: Addressing potentially negative consequences and outcomes for affected populations				
Unacceptable	Less than acceptable	Acceptable/Good		Very Good
1 2	3 4	5 6		7 8
There has been no apparent effort to address what could be serious negative consequences from the research process or results. The researchers appear to have been insensitive to this aspect of the research.	The research was sensitive to this issue. Some efforts were made to address what could turn into negative consequences or outcomes, but they were not as comprehensive or thorough as they should have been. Informed consent was not adequately assured, and coercion of vulnerable populations was not adequately avoided	The research was sensitive to this issue. Appropriate and timely measures have been taken in almost all instances to eradicate or mitigate foreseeable negative consequences or outcomes of the research. Measures have been taken to ensure compliance with the free, prior and informed consent processes and privacy of research participants. There is no sign of coercion of a vulnerable person, community or population		Appropriate and timely measures have been taken to eliminate or mitigate foreseeable negative consequences or outcomes of research. There was a systematic effort by the research team to mitigate negative consequences and outcomes. Measures have been taken to ensure participants’ free, prior and informed consent and to ensure their privacy. There are no signs of coercion of a vulnerable person, community or population.
Sub dimension 2.2: inclusiveness				
Unacceptable	Less than acceptable	Acceptable/Good	Very Good	
1 2	3 4	5 6	7 8	
Relevant selection processes and the prioritization and safeguarding of vulnerable or marginalized communities has not received sufficient attention in the research design and execution.	Inclusiveness has been partially addressed in the research design, execution and findings. Weaknesses remain, e.g., in selection processes, and/or the prioritization and safeguarding of vulnerable or marginalized communities demand more attention.	Inclusiveness has been appropriately addressed in research design, execution and findings. A few opportunities remain to strengthen selection processes, and/or the prioritization and safeguarding of vulnerable or marginalized communities.	Inclusiveness has been intentionally and systematically addressed in the research design, execution and findings. There are no weaknesses in relevant selection processes, and/or the prioritization and safeguarding of vulnerable or marginalized communities	
Sub dimension 2.3: Gender				
Unacceptable	Less than acceptable	Acceptable/Good	Very Good	
1 2	3 4	5 6	7 8	
The research was gender blind	*(Based on the category selected in the 1*^*st*^* column)*	*(Based on the category selected in the 1*^*st*^* column)*	*(Based on the category selected in the 1*^*st*^* column)*	
- Gender considerations were not included in the research questions or Objectives	Gender was considered in a limited way with notable weaknesses	Gender was adequately considered in most phases of the research cycle, and gender balance in participation.	Gender was fully considered in all aspects of the research cycle, and in participation	
Data collection did not register differences related disaggregated by sex	Data collection minimally accounted for few data were disaggregated by sex	- Gender was appropriately incorporated into the research questions and objectives		
- There was no consideration	Limited gender consideration was shown	Data collection accounted for differentiated situations	- Data collection accounted for differentiated situations related to gender	
1 2	3 4	5 6	7 8	
Engagement with appropriate contexts has been neglected during the research process. Several major weaknesses can be found, related to how research needs and questions were identified, communities or populations engaged, contexts and knowledge systems considered, and benefits from the research process assured.	Contexts and engagement have been considered during the research process, but some weaknesses remain related to how research needs and questions were identified, communities, stakeholders or populations engaged, contexts and knowledge systems considered, and/or local benefits from the research process assured	Context and engagement have been appropriately considered in the research process. Few, if any, minor weaknesses remain related to how research needs and questions were identified, communities, stakeholders or populations engaged, contexts and knowledge systems considered, or stakeholder benefits from the research process assured.	Context and engagement have been carefully and systematically considered in the research process. Research needs and questions were clearly identified, communities, stakeholders or populations effectively engaged, contexts and knowledge systems considered and respected, and stakeholder benefits from the research process assured	

Dimension 3: research importance			
Sub dimension 3.1: Originality			
Unacceptable	Less than acceptable	Acceptable/Good	Very Good
1 2	3 4	5 6	7 8
The research fails to build on and extend on existing knowledge. It does not break new ground or make improvements in existing technologies and/or methods	The research marginally adds to what is already known in the field. The research is not innovative and is not well connected to what is already known.	The research presents fresh ideas, brings an innovative approach to solving existing challenges, and/or deals with a new, emerging issue worth pursuing. It challenges taken-for-granted assumptions, builds on existing knowledge and is well connected to what is already known	The research is innovative and ground breaking. It builds on existing knowledge in a substantive way, making significant advancements to technologies and techniques
Sub dimension 3.2: Relevance			
Unacceptable	Less than acceptable	Acceptable/Good	Very Good
1 2	3 4	5 6	7 8
The research does not contribute to a key development priority, or an emerging area that might demand solutions in the foreseeable future. Justification for the work is absent or unconvincing.	The research makes little contribution to a key development priority or an emerging area that might demand solutions in the foreseeable future. A justification for this area of work is not well substantiated.	The research contributes to a key development priority, or an emerging area of some significance that might demand solutions in the near future. This area of work is justified.	The research makes an important contribution towards a key development priority, or an important emerging area that is highly likely to demand solutions in the near future. This area of work is well justified

Dimension 4: positioning for use			
Sub dimension:4.1 Knowledge accessibility and sharing			
Unacceptable	Less than acceptable	Acceptable/Good	Very Good
1 2	3 4	5 6	7 8
The research was not initiated and conducted with use in mind, i.e., no evidence of understanding of the context(s) within which the results are likely to be used; no evidence of stakeholder or user mapping. There has been no attention or engagement to making research findings available in formats and through mechanisms suited to well-targeted audiences. Potential users will struggle to know about and access these knowledge products.	There was insufficient effort to map, understand and engage stakeholders or key potential user groups, and limited engagement with understanding the larger context within which they operate. Insufficient attention has been paid to making research findings available in appropriate formats and through appropriate mechanisms to well-targeted potential user groups.	The project research mapped, understood and engaged stakeholders and potential user groups. Researchers appear to have a credible understanding of the context within which key potential users/user groups operate. Research findings were made available to different potential user groups in user-friendly formats.	The research was initiated and conducted with use in mind, and with an emphasis on engaging with the contexts of potential users. The research included sophisticated/highly differentiated stakeholder mapping and engagement. Research findings were appropriately available to well- targeted and influential potential user groups in highly accessible and user-friendly formats. Mechanisms for use have been explored

6, and

**Table 7:** Data collection template for capturing the SDGs interaction based on the Nilsson framework (Nilsson et al. 2016)

SDG/target	Indicators	Type of interaction and score	Direction of the relationship	Explanation/motivation
Explanation: Each project should list the SDG and targets that the project is addressing	Explanation: Each project should list the specific indicators that the project is addressing	Explanation: Positive interaction Indivisible (e.g. achievement on one goal automatically linked to achievement of another goal) Score: + 3 Reinforcing (e.g. achievement on one goal aids the achievement of another goal) Score: + 2 Enabling (e.g. achievement on one goal creates conditions that further another goal) Score: + 1 No significant interaction Consistent (e.g. no significant interactions between two targets achievements) Score: ± 0 Negative interaction Constraining (e.g. achievement on one goal limits options on another goal) Score: − 3 Counteracting (e.g. achievement on one goal makes clashes with another goal) Score: − 2 Cancelling (e. e.g. achievement on one goal automatically leads to a negative impact on another goal) Score: − 1	Explanation: Unidirectional (e.g. Objective A affect B, but B does not affect A) Bidirectional (e.g. A affects B, and B affects A) Interactions can also be symmetrical (where the impact is similar in type and strength) or, more commonly, asymmetrical, where A affects B more, or in different ways, compared to how B affects) Circular (e.g. in a circular relationship A affects B, which affects C, which in turn affects A) Multi-directionality** (**A affects B, C, D)	Explanation: Provide any additional reasons for your answers

7.
